# Supplementary material for: Patterns of Intron Gain and Loss in Fungi
Source: PLoS Biol. 2004 Nov 30;2(12):e422. doi: 10.1371/journal.pbio.0020422 (PMC532390; doi:10.1371/journal.pbio.0020422)
Supplement: Table S1 — Also available at http://genes.mit.edu/NielsenEtAl/. (4.3 MB ZIP). [file pbio.0020422.st001.zip › NielsenEtAl/html/256.html]

AN2930.1.NCU03283.1.MG00550.1.FG07057.1


```
 CLUSTAL W (1.82) Multiple Sequence Alignments - Introns Inserted


Sequence 1: NCU03283.1	230 aa
Sequence 2: MG00550.1	230 aa
Sequence 3: FG07057.1	238 aa
Sequence 4: AN2930.1	229 aa
Alignment Length: 238 aa
Number Identitical Residues: 118 aa
Alignment Score (without introns) 5938


MG00550.1 	--------MATLRNARKV~VCIGRNYA~DHIAELNSAKPKQPFFFLKPPSSIVAPGAGPV
NCU03283.1	--------MASLKKASKV~VCIGRNYA2DHITELNSAKPKQPFFFLKPPSSIILPGEGPV
FG07057.1 	MANLAPQASSSLKQAGKV~ICIGRNFA2EHIAELNSAKPKQPFFFLKPTSSIVLPGEGAV
AN2930.1  	-------MAASIKSNCRK0LTLDR--S~DHIAELNNTKPKQPFFFLKPSSSILCPGEGPV
          	         ::::.  :  : :.*  : :**:***.:***********.***: ** *.*

MG00550.1 	IRPKGVDLHYEVELALIIGKEVKNLKADDDQGALDAIES1YALSIDMTARNVQNEAKKKG
NCU03283.1	IRPRGVDMHYEVELALILGKRVRDHDASDLQGALDAIDS1YALSIDMTARNVQNEAKKKG
FG07057.1 	LRPKGIEMHYEVELALVIGKLTKDLQASDVQGAMDAVKA1YAVAIDFTARNAQNEAKKKG
AN2930.1  	LRPKGTSLHYEVELALVMGKKVRDLDPNDDKGALDAIQS1YLLAIDMTARNVQDEAKKKG
          	:**:* .:********::** .:: ...* :**:**:.: * ::**:****.*:******

MG00550.1 	LPWDIAKGFDTFLPFSNIIPKSAIPDPHNVELYLSVNGEVRQQDSTNLLLFRIPRVLADI
NCU03283.1	LPWDIAKGFDTFLPMSKLIEKSRIQDPHNIELYLKVNDEVRQNDSTNLMLFQIPRILSDI
FG07057.1 	LPWSIAKGFDTFLPMSNIIPKTAIQDPHNVELFLQLNGETKQQGSTNLMIYQIPRILSDI
AN2930.1  	LPWTIAKGFDTFLPISQEILKSQIPDPHNAFLRLSVNSQQRQADSSGLMLYRIPRLLAEI
          	*** **********:*: * *: * ****  * *.:*.: :* .*:.*::::***:*::*

MG00550.1 	SSVMTLEKGDIVLTGTPKGVGPVVPGDVMRAGIRVNGKEIEEARIEIPVEESTSDYFYKE
NCU03283.1	SKVMTLEAGDIVLTGTPKGVGPVVPGDVMRAGIRVDGKEIEEAKIEIAVEESTRAYEYAE
FG07057.1 	SKVMTLHPGDIVLTGTPAGVGPAAPGDVVRAGLRIDGKEVEEGAIEIPVEESPSSYTFSE
AN2930.1  	SRVMTLEKGDLVLTGTPKGVGEVKSGDVMKASIEVDGKEIEQGRIEVEVQDREGRYEYCE
          	* ****. **:****** *** . .***::*.:.::***:*:. **: *::    * : *

MG00550.1 	T
NCU03283.1	T
FG07057.1 	T
AN2930.1  	T
          	*
```
